# Peripheral T lymphocyte and immunocyte subset dynamics: markers of neoadjuvant therapy outcomes in esophageal squamous cell carcinoma

**DOI:** 10.3389/fimmu.2023.1320282

**Published:** 2023-12-21

**Authors:** Xin Nie, Shuya He, Xinming Nie, Changding Li, Kunyi Du, Wenwu He, Zhiyu Li, Kunhan Ni, Simiao Lu, Chenghao Wang, Kangning Wang, Yan Miao, Longlin Jiang, Jiahua Lv, Guangyuan Liu, Qiang Fang, Lin Peng, Wenguang Xiao, Qifeng Wang, Dongsheng Wang, Yongtao Han, Xuefeng Leng

**Affiliations:** ^1^ Department of Thoracic Surgery, Sichuan Cancer Research Center for Cancer, Sichuan Cancer Hospital and Institute, Sichuan Cancer Center, Affiliated Cancer Hospital of University of Electronic Science and Technology of China, Chengdu, Sichuan, China; ^2^ Department of Clinical Laboratory, Sichuan Cancer Research Center for Cancer, Sichuan Cancer Hospital and Institute, Sichuan Cancer Center, Affiliated Cancer Hospital of University of Electronic Science and Technology of China, Chengdu, Sichuan, China; ^3^ Department of Radiation Oncology, Radiation Oncology Key Laboratory of Sichuan Province, Sichuan Cancer Research Center for Cancer, Sichuan Cancer Hospital and Institute, Sichuan Cancer Center, Affiliated Cancer Hospital of University of Electronic Science and Technology of China, Chengdu, Sichuan, China

**Keywords:** esophageal squamous cell carcinoma, neoadjuvant chemoradiotherapy, lymphocyte subsets, peripheral blood cell, major pathological response

## Abstract

**Purpose:**

In patients with resectable esophageal squamous cell carcinoma (ESCC), neoadjuvant therapy increased the curative resection rate, disease-free survival, and overall survival for patients with resectable ESCC. However, the efficacy of neoadjuvant therapy varies among different patients. We aim to compare the differences in the characteristics of peripheral blood T lymphocyte subsets before and after neoadjuvant therapy in patients with different curative efficacy.

**Method:**

This study enrolled 266 ESCC patients who received neoadjuvant therapy and esophagectomy from August 2018 to August 2022. The postoperative pathological results divided patients into the major pathological response (MPR) and non-MPR groups. Compare the differences in peripheral blood T lymphocyte subsets and analyze the trend of changes in T lymphocyte subsets at different phases of treatment. Propensity score matching was used to reduce the influence of potential confounding factors.

**Results:**

Prior to the neoadjuvant therapy, particularly before the second cycle, the MPR group exhibited significantly higher ratios of CD4/CD8 (*P*=0.009) and helper T cells (TH ratio, *P*=0.030) compared to the non-MPR group. In contrast, the suppressor T cell ratio (TS ratio) was lower (*P*=0.016) in the MPR group. The difference in peripheral blood lymphocyte subsets between the two groups of patients who underwent neoadjuvant chemoradiotherapy is significant.

**Conclusion:**

In peripheral blood, T lymphocyte subsets varied significantly based on the effectiveness of neoadjuvant treatment. Prior to the second cycle of neoadjuvant therapy, a higher CD4/CD8 and TH ratio, coupled with a decreased TS ratio, might suggest enhanced treatment outcomes.

## Introduction

Esophageal cancer (EC) has the seventh highest incidence among 36 human cancers and is the sixth leading cause of cancer-related death globally (1, 2). In China, esophageal squamous cell carcinoma (ESCC) is the main subtype, accounting for more than 90% of all cases (3). Neoadjuvant therapy plus esophagectomy is the standard treatment strategy for resectable EC, and it could markedly prolong overall survival (OS) and improve prognosis (4, 5). The results of the CROSS trial indicated the sensitivity of ESCC to neoadjuvant chemoradiotherapy, with a pCR rate of 49.0% after neoadjuvant chemoradiotherapy (6). After that, The clinical trial of NEOCRTEC5010 in China revealed that the pCR rate of patients with ESCC after receiving neoadjuvant chemoradiotherapy was about 45.2%, and the disease-free survival (DFS) was significantly prolonged compared to the surgery alone group (5). Nevertheless, some patients with EC may not benefit from neoadjuvant therapy, and some may even progress during the neoadjuvant therapy process.

The immune system function is one of the most critical factors affecting the prognosis of cancer treatment (7). The immune function of cancer patients is mainly composed of T lymphocyte subsets, B cells, and natural killer (NK) cells. Lymphocytes have immune recognition functions and are essential cellular components in the immune response. T lymphocytes can be further divided into CD4+T lymphocytes (CD3+CD4+, Helper T cells, TH) and CD8+T lymphocytes (CD3+CD8+, Suppressor T cells, TS) based on their surface markers (8). Monitoring lymphocyte subpopulation levels is crucial for guiding the combination therapy of multiple cancers, making it possible to link immune status with treatment response in cancer patients receiving neoadjuvant therapy (9). Most studies on the cellular immune status of patients with EC are based on T lymphocyte subsets and NK cells (10–13). The detection of peripheral blood in the above lymphocyte subsets makes it more convenient to obtain the relevant information of patients.

Herein, the primary objective is to compare the differences in peripheral blood T cell characteristics among patients with different neoadjuvant treatment efficacy, concurrently, our secondary goal is to observe the trend of changes in peripheral blood T cells at different treatment stages.

## Methods

### Patients

Patients with EC who underwent neoadjuvant therapy and surgery at Sichuan Cancer Hospital and Institute from August 2018 to August 2022 (retrospective data were available until July 1, 2020, and prospective data thereafter) were recruited into the study based on the Sichuan Cancer Hospital & Institute Esophageal Cancer Case Management Database (SCCH-ECCM Database). The collected information includes demographic data, pre-treatment clinical TNM (cTNM) staging data, neoadjuvant chemotherapy regimen, and pathological results. Prior to treatment, cTNM staging was verified using endoscopy, endoscopic ultrasonography, and computed tomography (CT). Positron emission tomography computed tomography (PET-CT) was not a routine examination, it was determined based on the patient’s situation. TNM staging is based on the American Joint Commission on Cancer (AJCC) Cancer Staging Manual, 8th edition (14).

The inclusion criteria were: (1) Histologically proven resectable ESCC; (2) First treatment at our hospital; (3) Precise pathological diagnoses after surgery. Exclusion criteria: (1)Patients with diseases that seriously impair immune function, such as acquired immunodeficiency syndrome, systemic lupus erythematosus, etc.; (2) There is a clear history of infection recently; (3) Recently used immune enhancers; (4) Having a history of other malignant tumors or mixed pathological types; (5) Incomplete laboratory data; All procedures performed in this study involving human participants were following the Declaration of Helsinki (as revised in 2013). The study was authorized by the Ethics Committee (EC) for Medical Research and New Medical Technology of Sichuan Cancer Hospital (SCCHEC-02-2017-043, ClinicalTrials.gov ID: NCT04440332). All the patients provided written informed consent.

### Treatment procedures and efficacy assessments

All ESCC patients underwent neoadjuvant therapy, which encompassed neoadjuvant chemoradiotherapy, chemotherapy, and radiotherapy. The predominant chemotherapy regimens were paclitaxel combined with either carboplatin (TC) or cisplatin (TP), with a total of two cycles administered. Each treatment cycle was 3 weeks. Two radiation oncologists with >10 years of experience co-developed the radiation treatment plans. The radiation doses were 40.0 Gy, with a fraction size of 1.8–2.0 Gy delivered once daily, five times per week. Surgeries were conducted as scheduled by McKeown approach 4-8 weeks following the end of neoadjuvant therapy. Blood samples were taken at five distinct intervals: prior to the first cycle and second cycle of neoadjuvant therapy, within a week before and after the surgery, and during the first post-operative follow-up. The proportions of CD4+, CD8+, and CD4/CD8 on the surface of peripheral blood T lymphocytes were assessed using flow cytometry.

After a review of the primary tumors and lymph nodes by experienced pathologists, all patients who underwent surgery had the final available pathological stage (ypTNM). The criteria for pathological assessment were based on the 8th edition of the TNM staging system. Conventional hematoxylin and eosin staining were used to detect the percentage of remaining surviving tumors at the primary site. Tumors exhibiting less than 10% residual tumor cells are classified as demonstrating major pathological reactions (MPR). Based on postoperative pathological findings, patients were stratified into MPR and non-MPR cohorts. The disparities in peripheral blood T lymphocyte subsets between these cohorts were assessed prior to neoadjuvant intervention. A dynamic analysis was conducted to elucidate the evolving trends of these subsets from pre-neoadjuvant therapy through post-esophagectomy.

### Statistical analysis

Categorical variables between the two groups were compared using the Chi-square test or Fisher’s exact test. Due to the non-normal distribution, the Wilcoxon Mann-Whitney test was used to compare the main results between the two groups. Linear mixed-effect models were used to assess differences between the two groups and whether the differences in changes over time between different groups were significant. Patient group, time (detection time point of T lymphocytes), and the interaction between the patient group and time are designated as fixed effects. The subjects and time were designated as random effects. Use the maximum likelihood estimation method to estimate the fixed effects and intercepts of all dependent variables and the random effects of the subjects. Unbalanced covariates were adjusted by performing propensity score matching (PSM) to create two comparable groups. A logit model was used to estimate patient propensity scores that included age, sex, smoking history, drinking history, clinical stage, tumor location, and neoadjuvant therapy for the cohort of PSM. Nearest neighbor matching (1:1) was performed without replacement based on a prespecified caliper width (0.02) to match patients in the MPR and non-MPR groups. A two-sided p-value of less than 0.05 was considered statistically significant. All statistical analyses were performed using the GraphPad Prism 8 (GraphPad Software Inc., San Diego, CA, USA) or statistical package SPSS (26.0 SPSS Inc., Chicago, IL, USA).

## Results

### Patient characteristics

A total of 266 patients with ESCC who received neoadjuvant therapy and esophagectomy were enrolled in this study. The clinical and pathological characteristics of the patients are shown in **Table 1**. Of the resected primary tumors, 47.4% (126/266) demonstrated major pathological responses (MPR). In the MPR group, the majority (54.0%) had middle thoracic ESCC, whereas in the non-MPR group, 50.7% were diagnosed with lower thoracic ESCC (*P*<0.01). Ninety-six (76.2%) patients in the MPR group received neoadjuvant chemoradiotherapy. In the non-MPR group, patients receiving neoadjuvant chemoradiotherapy, neoadjuvant chemotherapy, and neoadjuvant radiotherapy accounted for 50%, 48.6%, and 1.4%, respectively (*P*<0.01). After PSM, there were 174 remaining patients, 87 in each group, with no significant difference at baseline.

**Table 1:** Patient characteristics at baseline based on pathological response.

Characteristics	Before PSM				After PSM			
	All patients N = 266	Patients with major pathological response N = 126	Patients without major pathological response N = 140	P-value	All patients N = 174	Patients with major pathological response N = 87	Patients without major pathological response N = 87	P-value
Age (Mean ± SD)	61.0 ± 7.1	62.0 ± 6.8	60.1 ± 7.4	0.036*	61.9 ± 7.1	61.9 ± 7.0	61.8 ± 7.2	0.940
Gender Male Female	227 (85.3%) 39 (14.7%)	106 (84.1%) 20 (15.9%)	121 (86.4%) 19 (13.6%)	0.596	155 (89.1%) 19 (10.9%)	77 (88.5%) 10 (11.5%)	78 (89.7%) 9 (10.3%)	0.808
Smoking history Yes No	181 (68.0%) 85 (32.0%)	86 (68.3%) 40 (31.7%)	95 (67.9%) 45 (32.1%)	0.468	122 (70.1%) 52 (29.9%)	62 (71.3%) 25 (28.7%)	60 (69.0%) 27 (31.0%)	0.740
Drinking history Yes No	167 (68.2%) 99 (37.2%)	76 (60.3%) 50 (39.7%)	91 (65.0%) 49 (35.0%)	0.430	115 (66.1%) 59 (33.9%)	59 (67.8%) 28 (32.2%)	56 (64.4%) 31 (35.6%)	0.631
Clinical Stage (8th edition) I II III IVa	4 (1.5%) 40 (15.0%) 168 (63.2%) 54 (20.3%)	3 (2.4%) 18 (14.3%) 84 (66.7%) 21 (16.7%)	1 (0.7%) 22 (15.7%) 84 (60.0%) 33 (23.6%)	0.361	3 (1.7%) 26 (14.9%) 114 (65.5%) 31 (17.8%)	2 (2.3%) 12 (13.8%) 59 (67.8%) 14 (16.1%)	1 (1.1%) 14 (16.1%) 55 (63.2%) 17 (19.5%)	0.821
Tumor location Upper Middle Lower	35 (13.2%) 123 (46.2%) 108 (40.6%)	21 (16.7%) 68 (54.0%) 37 (29.4%)	14 (10.0%) 55 (39.3%) 71 (50.7%)	<0.01*	21 (12.1%) 85 (48.9%) 68 (39.1%)	9 (10.3%) 45 (51.7%) 33 (37.9%)	12 (13.8%) 40 (46.0%) 35 (40.2%)	0.684
Neoadjuvant Chemoradiotherapy Chemotherapy Radiotherapy	166 (62.4%) 96 (36.1%) 4 (1.5%)	96 (76.2%) 28 (22.2%) 2 (1.6%)	70 (50%) 68 (48.6%) 2 (1.4%)	<0.01*	119 (68.4%) 53 (30.5) 2 (1.1%)	59 (67.8%) 26 (29.9%) 2 (2.3%)	60 (69.0%) 27 (31.0%) 0 (0%)	0.591

* P-value < 0.05.

PSM, propensity score matching.

### T lymphocyte subsets characteristics before surgery

**Figure 1:**
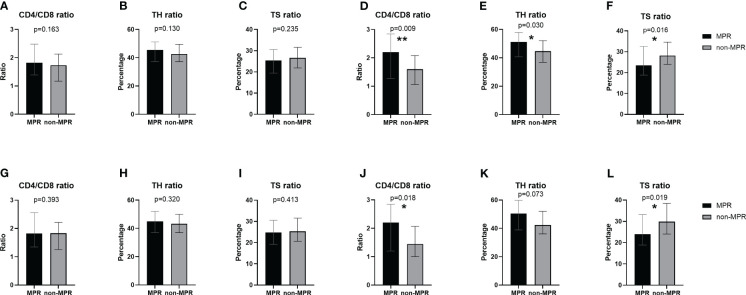
Comparison of peripheral blood T lymphocyte subsets between the MPR and non-MPR groups. The ratio of CD4/CD8 **(A, G)**, TH **(B, H)**, TS **(C, I)** before the first cycle of neoadjuvant therapy and the ratio of CD4/CD8 **(D, J)**, TH **(E, K)**, TS **(F, L)** before the second cycle of neoadjuvant therapy before and after PSM. *P < 0.05; **P < 0.01, with Wilcoxon Mann-Whitney test. MPR, major pathological reaction; PSM, propensity score matching; TH, helper T cells; TS, suppressor T cells.

Prior to the initiation of neoadjuvant therapy, no marked discrepancies were observed in the peripheral blood lymphocyte subsets between the MPR and non-MPR groups (**Figures 1A–C**). Nonetheless, before the second cycle of neoadjuvant therapy, the MPR cohort exhibited a notably elevated CD4/CD8 ratio compared to the non-MPR cohort (*P*=0.009, **Figure 1D**). Similarly, the TH ratio in the MPR group surpassed that of the non-MPR group (*P*=0.03, **Figure 1E**). Conversely, the TS ratio manifested a decline in the MPR group (*P*=0.016, **Figure 1F**). After PSM, there was no significant difference between the two groups in T lymphocyte subsets before the first cycle of neoadjuvant therapy (**Figures 1G–I**). Before the second cycle, the CD4/CD8 ratio was significantly higher (*P*=0.018, **Figure 1J**). There was no significant difference in the TH ratio (**Figure 1K**). The TS ratio was considerably lower in the MPR group (*P*=0.019, **Figure 1L**).

### Longitudinal changes of T lymphocyte

**Figure 2:**
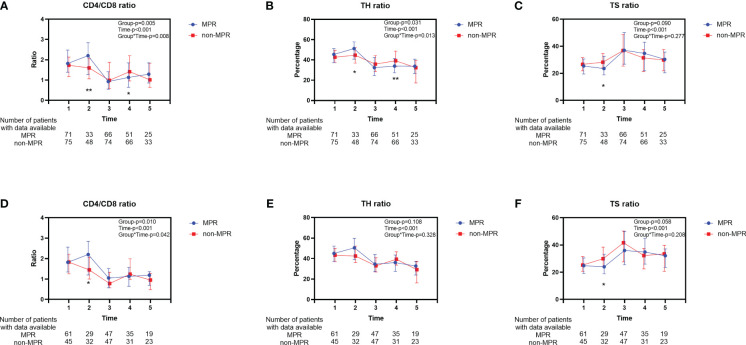
Longitudinal changes of peripheral blood T lymphocyte subsets between the MPR and non-MPR groups. The ratio of CD4/CD8 **(A, D)**, TH **(B, E)**, TS **(C, F)** for all patients before and after PSM. Time 1, 2, 3, 4, 5 represented prior to the first cycle of neoadjuvant therapy, before the second cycle of neoadjuvant therapy, within 7 days before surgery, within 7 days after surgery, and during the first follow-up, respectively. Error bars represent interquartile range. *P < 0.05; **P < 0.01, with Wilcoxon Mann-Whitney test. MPR, major pathological reaction; PSM, propensity score matching; TH, helper T cells; TS, suppressor T cells.

Significant alterations in the peripheral blood T lymphocyte subsets were observed across various treatment phases in patients with ESCC (Time-*P*<0.001, **Figure 2**). During the second cycle of neoadjuvant therapy, the MPR cohort exhibited an elevation in the CD4/CD8 ratio (**Figure 2A**) and TH ratio (**Figure 2B**) relative to the initial cycle. Subsequently, these ratios markedly declined, with the trends diverging across different treatment stages. Conversely, the TS ratio demonstrated an inverse pattern (**Figure 2C**). The trend in TS ratio changes did not display a significant difference between the two groups (Group-*P*=0.09, **Figure 2C**). After PSM, the movement of changes in T lymphocyte subsets was similar to before PSM (**Figures 2D–F**). However, except for the difference of TS ratio at the second time point, there was no statistically significant trend in the changes of TH ratio (Group-*P*=0.108, **Figure 2E**) and TS ratio (Group-*P*=0.058, **Figure 2F**) between the two groups throughout the entire treatment process. Furthermore, no difference was observed in the percentage of T lymphocytes between the two groups before and after PSM (**Supplementary Figures 1A, B**).

### Subgroup analysis

**Figure 3:**
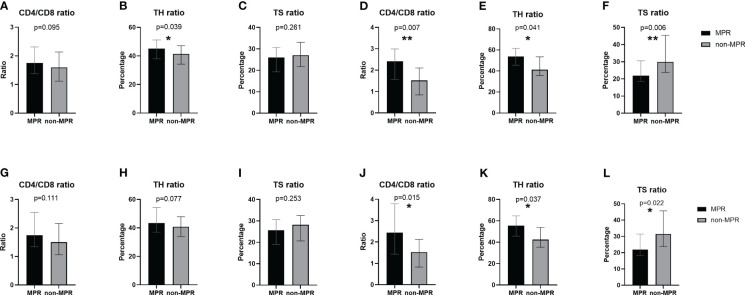
Comparison of peripheral blood T lymphocyte subsets between the MPR and non-MPR groups. The ratio of CD4/CD8 **(A, G)**, TH **(B, H)**, TS **(C, I)** before the first cycle of neoadjuvant chemoradiotherapy and the ratio of CD4/CD8 **(D, J)**, TH **(E, K)**, TS **(F, L)** before the second cycle of neoadjuvant chemoradiotherapy before and after PSM. *P < 0.05; **P < 0.01, with Wilcoxon Mann-Whitney test. MPR, major pathological reaction; PSM, propensity score matching; TH, helper T cells; TS, suppressor T cells.

**Figure 4:**
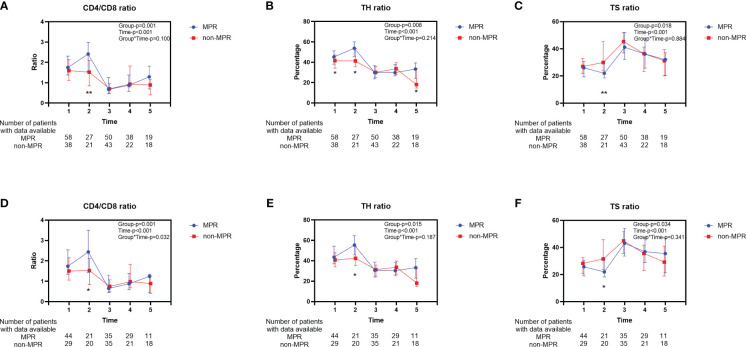
Longitudinal changes of peripheral blood T lymphocyte subsets between the MPR and non-MPR groups. The ratio of CD4/CD8 **(A, D)**, TH **(B, E)**, TS **(C, F)** for patients who received neoadjuvant chemoradiotherapy before and after PSM. Time 1, 2, 3, 4, 5 represented prior to the first cycle of neoadjuvant therapy, before the second cycle of neoadjuvant therapy, within 7 days before surgery, within 7 days after surgery, and during the first follow-up, respectively. Error bars represent interquartile range. *P < 0.05; **P < 0.01, with Wilcoxon Mann-Whitney test. MPR, major pathological reaction; TH, helper T cells; TS, suppressor T cells.

The characteristics of 166 patients who underwent neoadjuvant chemoradiotherapy are shown in **Table 2**. For the 96 patients in the MPR group, more than half (53.1%) had middle thoracic ESCC, whereas, in the non-MPR group, 41.4% were diagnosed with middle thoracic ESCC and 47.1% were diagnosed with lower thoracic ESCC (*P*=0.023). Before initiating neoadjuvant therapy, the MPR group exhibited an elevated peripheral blood TH ratio compared to the non-MPR group. Still, there was no significant difference in CD4/CD8 and TS ratio (**Figures 3A–C**). Before the second cycle of neoadjuvant therapy, the peripheral blood CD4/CD8 (*P*=0.007, **Figure 3D**) and TH ratios (*P*=0.041, **Figure 3E**) in the MPR group were significantly superior to those in the non-MPR group. Conversely, the TS ratio of the MPR group was markedly lower than that in the non-MPR group (*P*=0.006, **Figure 3F**). After PSM, there was no significant difference in peripheral blood lymphocyte subsets between the MPR and the non-MPR groups before the first treatment cycle (**Figures 3G–I**). Furthermore, the ratio of CD4/CD8 (*P*=0.015, **Figure 3J**), TH (*P*=0.037, **Figure 3K**), and TS (*P*=0.022, **Figure 3L**) before the second cycle of neoadjuvant chemoradiotherapy were still significant differences after PSM. Throughout different treatment phases, there were significant changes in peripheral blood T lymphocyte subsets (Time-*P*<0.001). Besides, distinct differences were observed in the trends of CD4/CD8 ratio (Group-P=0.001, **Figure 4A**), TH ratio (Group-P=0.008, **Figure 4B**), and TS ratio (P=0.018, **Figure 4C**) between the two patient cohorts. And the trend of changes in T lymphocyte subsets was similar to before PSM (**Figures 4D–F**).

**Table 2:** Patient characteristics at baseline based on pathological response (Subgroup : Neoadjuvant Chemoradiotherapy).

Characteristics	Before PSM				After PSM			
	All patients N = 166	Patients with major pathological response N = 96	Patients without major pathological response N = 70	P-value	All patients N = 118	Patients with major pathological response N = 59	Patients without major pathological response N = 59	P-value
Age (Mean ± SD)	61.1 ± 7.1	61.2 ± 7.0	60.9 ± 7.2	0.759	60.7 ± 7.3	61.1 ± 7.1	60.1 ± 7.6	0.517
Gender Male Female	143 (86.1%) 23 (13.9%)	80 (83.3%) 16 (16.7%)	63 (90.0%) 7 (10.0%)	0.220	105 (89.0%) 13 (11.0%)	53 (89.8%) 6 (10.2%)	52 (88.1%) 7 (11.9%)	0.769
Smoking history Yes No	113 (68.1%) 53 (31.9%)	61 (63.5%) 35 (36.5%)	46 (65.7%) 24 (34.3%)	0.773	88 (74.6%) 30 (25.4%)	44 (74.6%) 15 (25.4%)	44 (74.6%) 15 (25.4%)	1.000
Drinking history Yes No	109 (65.7%) 57 (34.3%)	67 (69.8%) 29 (30.2%)	48 (68.6%) 22 (31.4%)	0.886	86 (72.9%) 32 (27.1%)	43 (72.9%) 16 (27.1%)	43 (72.9%) 16 (27.1%)	1.000
Clinical Stage (8th edition) I II III IVa	4 (1.5%) 13 (7.8%) 114 (68.7%) 35 (21.1%)	3 (3.1%) 9 (9.4%) 65 (67.7%) 19 (19.8%)	1 (1.4%) 4 (5.7%) 49 (70.0%) 16 (22.9%)	0.745	1 (0.8%) 8 (6.8%) 81 (68.6%) 28 (23.7%)	0 (0%) 4 (6.8%) 41 (69.5%) 14 (23.7%)	1 (1.7%) 4 (6.8%) 40 (67.8%) 14 (23.7%)	1.000
Tumor location Upper Middle Lower	27 (16.3%) 80 (48.2%) 59 (35.5%)	19 (19.8%) 51 (53.1%) 26 (27.1%)	8 (11.4%) 29 (41.4%) 33 (47.1%)	0.023*	14 (11.9%) 60 (50.8%) 44 (37.3%)	6 (10.2%) 33 (55.9%) 20 (33.9%)	8 (13.6%) 27 (45.8%) 24 (40.7%)	0.535

* P-value < 0.05.

PSM, propensity score matching.

## Discussion

Neoadjuvant chemoradiotherapy is the standard preoperative treatment regimen for locally advanced resectable esophageal cancer, with most patients benefiting from it and significantly improving overall survival (15). However, some patients cannot benefit from neoadjuvant therapy, and even the disease progresses during this treatment process. Therefore, finding reliable biomarkers and/or predictors to predict the efficacy of neoadjuvant therapy is very meaningful.

So far, some biomarkers have been considered as candidate predictors of neoadjuvant therapy sensitivity, such as miR-200c (16), Let-7 (17), Micro-RNAs (18), and p53 (19). The methods for detecting these biomarkers are complex, expensive, and low in accuracy. Therefore, they have not been widely used in clinical practice. There is still a need for convenient, accurate, and economical predictive factors to identify patients who are more likely to benefit from neoadjuvant therapy. Previous studies have predicted the efficacy of neoadjuvant therapy or the prognosis of patients through peripheral blood indicators. Ji et al. found that in patients with advanced ESCC, prechemotherapy neutrophil-to-lymphocyte ratio (NLR) is an independent prognostic factor for overall survival (20). Moreover, NLR and platelet-to-lymphocyte ratio (PLR) can provide a simple serum test to evaluate the effectiveness of neoadjuvant therapy (21). In a retrospective cohort study of non-small cell lung cancer, high baseline absolute CD4+T lymphocyte count helps extend progression-free survival (22). Lymphocyte subsets will change dramatically during neoadjuvant chemoradiotherapy, and these changes have predictive value for the response to neoadjuvant chemoradiotherapy (23). Lymphocytes account for approximately 30% of the total number of normal human white blood cells and are essential effector cells in anti-tumor immunity. The changes in lymphocyte subsets are closely related to the progression and prognosis of tumors (24).

In our investigation, we revealed the disparities in peripheral blood T lymphocyte subsets relative to the efficacy of neoadjuvant therapy, as well as the changing trend in these subsets across different treatment stages. Notably, patients with an elevated proportion of CD4/CD8 ratio in peripheral blood T lymphocytes tend to experience more favorable outcomes from neoadjuvant interventions. These patients typically present with a higher TH ratio and a lower TS ratio. However, there was no significant difference in the proportion of T lymphocytes between the two groups. Intriguingly, the MPR rate was higher in patients receiving neoadjuvant chemoradiotherapy (57.83%, 96/166), while the MPR rate was significantly lower in patients receiving neoadjuvant chemotherapy (29.17%, 28/96). Given these findings, a focused subgroup analysis on the neoadjuvant chemoradiotherapy patients was undertaken, revealing even more pronounced difference in T lymphocyte subsets between the two groups. Furthermore, the lymphocyte subsets showed significant modifications across treatment stages, with the nature of these shifts varying based on the therapeutic efficacy.

This study also has some limitations. Firstly, our approach combined both retrospective and prospective designs, was conducted within a single center, and was limited by an insufficient follow-up data set. To truly validate the significance and clinical implications of our findings, a comprehensive prospective, multi-center, large-scale clinical trial is essential. Secondly, the majority of patients exhibited varying extents of leukopenia during treatment, and necessitating the use of medications to elevate their white blood cell counts, which could have influenced the results of peripheral blood lymphocyte count after neoadjuvant therapy. To mitigate this, we assessed therapeutic efficacy by analyzing the relative proportion of various lymphocyte subsets. Lastly, a subset of patients received immunoenhancers, such as Thymosin Alpha-1 for Injection, during preoperative care, potentially influencing both the efficacy of the neoadjuvant therapy and composition of lymphocyte subsets. A meticulously planned prospective study is warranted for deeper insights.

In summary, peripheral blood T lymphocyte subsets exhibit no discernible differences prior to the initiation of neoadjuvant therapy. Yet, significant disparities emerge following the initial neoadjuvant therapy cycle, with distinct patterns evolving based on therapeutic outcomes. The characteristics of dynamics observed during the neoadjuvant treatment period offer valuable prognostic insights into the efficacy of neoadjuvant therapy for individual patients.

## Data availability statement

The raw data supporting the conclusions of this article will be made available by the authors, without undue reservation.

## Ethics statement

The studies involving humans were approved by The study was authorized by the Ethics Committee (EC) for Medical Research and New Medical Technology of Sichuan Cancer Hospital (SCCHEC-02-2017-043, ClinicalTrials.gov ID: NCT04440332). The studies were conducted in accordance with the local legislation and institutional requirements. The participants provided their written informed consent to participate in this study.

## Author contributions

XN: Writing – original draft, Writing – review & editing, Conceptualization, Data curation, Methodology, Software, Validation. SH: Writing – review & editing, Conceptualization, Data curation, Software, Validation. XMN: Writing – review & editing, Data curation, Supervision, Methodology, Software. CL: Writing – review & editing, Software, Data curation. KD: Writing – review & editing, Supervision, Data curation. WH: Writing – review & editing, Data curation, Supervision, Validation. ZL: Writing – review & editing, Methodology, Software, Data curation. KN: Writing – review & editing, Data curation, Supervision. SL: Writing – review & editing, Data curation, Software. CW: Writing – review & editing, Conceptualization, Validation. KW: Writing – review & editing, Data curation, Software. YM: Writing – review & editing, Software, Supervision. LJ: Data curation, Software, Writing – review & editing. JL: Data curation, Supervision, Writing – review & editing. GL: Writing – review & editing, Data curation, Software. QF: Writing – review & editing, Data curation, Methodology. LP: Writing – review & editing, Conceptualization, Supervision. WX: Writing – review & editing, Supervision, Software. QW: Writing – review & editing, Data curation, Methodology, Software. DW: Data curation, Validation, Writing – review & editing, Conceptualization, Methodology, Supervision. YH: Conceptualization, Methodology, Supervision, Validation, Writing – review & editing. XL: Writing – review & editing, Formal Analysis, Supervision, Validation, Conceptualization, Data curation, Methodology, Project administration, Software.

## Supplementary material

Supplementary Figure 1 Longitudinal changes of T lymphocyte **(A, B)** for all patients before and after PSM. Longitudinal changes of T lymphocyte **(C, D)** for patients who received neoadjuvant chemoradiotherapy before and after PSM. Time 1, 2, 3, 4, 5 represented before the first cycle of neoadjuvant therapy, before the second cycle of neoadjuvant therapy, within 7 days before surgery, within 7 days after surgery, and during the first follow-up, respectively. Error bars represent interquartile range. PSM, propensity score matching.
