# Supplementary figures and images for: Peripheral T lymphocyte and immunocyte subset dynamics: markers of neoadjuvant therapy outcomes in esophageal squamous cell carcinoma
Source: Front Immunol. 2023 Dec 21;14:1320282. doi: 10.3389/fimmu.2023.1320282 (PMC10764521; doi:10.3389/fimmu.2023.1320282)

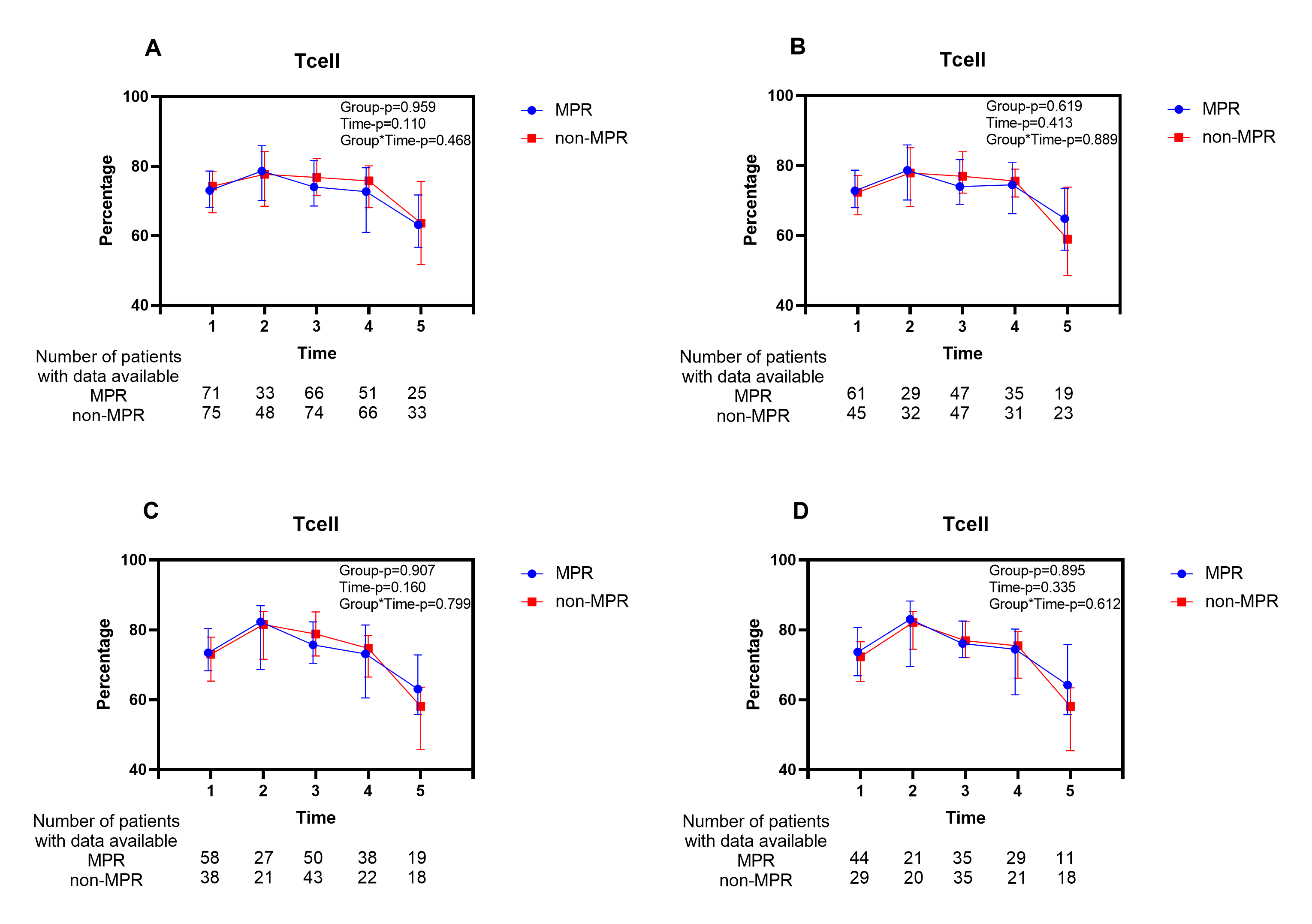

Supplement: Supplementary Figure 1 — Longitudinal changes of T lymphocyte (A, B) for all patients before and after PSM. Longitudinal changes of T lymphocyte (C, D) for patients who received neoadjuvant chemoradiotherapy before and after PSM. Time 1, 2, 3, 4, 5 represented before the first cycle of neoadjuvant therapy, before the second cycle of neoadjuvant therapy, within 7 days before surgery, within 7 days after surgery, and during the first follow-up, respectively. Error bars represent interquartile range. PSM, propensity score matching. [file Image_1.tif]
